# Supplementary material for: Synthesis and Characterization of Colistin-Functionalized Silica Materials for Rapid Capture of Bacteria in Water
Source: Molecules. 2022 Nov 28;27(23):8292. doi: 10.3390/molecules27238292 (PMC9739998; doi:10.3390/molecules27238292)
Supplement: Supplementary file 1 [file molecules-27-08292-s001.zip › molecules-2021721-supplementary.pdf]

Preparation of SiO_2@IPTS@ACA

The 0.5 g active bare silica gel was dispersed in 30 mL toluene, and then spiked with 500 μL (2 mmol) IPTS. The mixture was vortex mixed and reacted at 105℃ for 16h with reflux. The IPTS modified silica gel was repeated washing with DMF for 3 times, and then was added in 30 mL DMF with 10 mmol ACA. The reaction was occurred at 95℃ for 5h. The silica gel synthesized in the previous step was repeatedly washed by DMF and MES buffer (50 mM, pH 5.5±0.1) for 3 times. Finally, the synthesis of SiO_2@IPTS@ACA@CST was carried out by the steps of "2.3.3".

Preparation of SiO2@NH2@HDMI@ACA

The 0.5 g SiO2@NH2 was dispersed in 30 mL DMF, and then spiked with 400 μL (2.5 mmol) HMDI. The mixture was vortex mixed and reacted at 70℃ for 2h. The HDMI modified silica gel was purified via repeated washing with DMF for 3 times. and then was added in 30 mL DMF with 10 mmol ACA. The reaction was occurred at 70℃ for 12h. The silica gel synthesized in the previous step was repeatedly washed with DMF and MES buffer (50 mM, pH 5.5±0.1) for 3 times. Finally, SiO_2@NH_2@HDMI@ACA@CST was synthesized by the steps of "2.3.3".

Preparation of SiO2@NH2@COOH@ACA

The 0.5 g COOH modified silica gel was dispersed in 25 mL MES solution (50 mM, pH 5.5±0.1), and then spiked with 50 mg EDC·HCl and NHS. The mixture was vortex mixed and reacted at room 25 ℃ for 1 h. The modified silica gel was purified via repeated washing with MES solution for 3 times. and then was added in 25 mL MES solution with 10 mmol ACA and reaction was carried out at 25℃ for 2 h. The silica gel synthesized in the previous step was repeatedly washed with MES buffer and ultrapure water for 3 times. Finally, SiO2@COOH@ACA@CST was synthesized by the steps of "2.3.3".

The number of *E. coli* at various wet weigh was counted and their relationship was also evaluated. The number of E. coli cells is remarkably correlated with the wet weigh (y= 4.05×10^8 x, r^2 = 0.9965, n = 3). Consequently, the number of bacteria cells adsorbed by modified silica gel could be expressed as the wet weigh of *E. coli*.

Adsorption kinetics models

Pseudo-first-order and pseudo-second-order kinetic model were adopted to fit the dynamic adsorption model. The fitting equation of Pseudo-first-order kinetic model is shown in formula (3). Pseudo-first-order kinetic model was fitted by taking Q_t as the Y-axis and t as the X-axis. Parameters of Q_e and K_1 were calculated according to the fitting equation.

$$Q_t = Q_e(1 - e^{-k_1 t}) \qquad (1)$$

where Q_t ($\times10^{11}$ CFU/g) means adsorption capacity of *E. coli* at different time; Q_e means equilibrium adsorption capacity obtained by fitting and K_1 (min^{-1}) is Pseudo-first-order kinetic model rate constant.

Pseudo-second-order kinetic model was fitted by taking Q_t as the Y-axis and t as the X-axis. Parameters of Q_e and K_2 were calculated according to the fitting equation.

$$Q_t = \frac{k_2 {Q_e}^2 t}{1+k_2 Q_e t} \qquad (2)$$

where K_2 (g $\times10^{11}$ CFU^{-1} min^{-1}) is Pseudo-first-order kinetic model rate constant

Figures

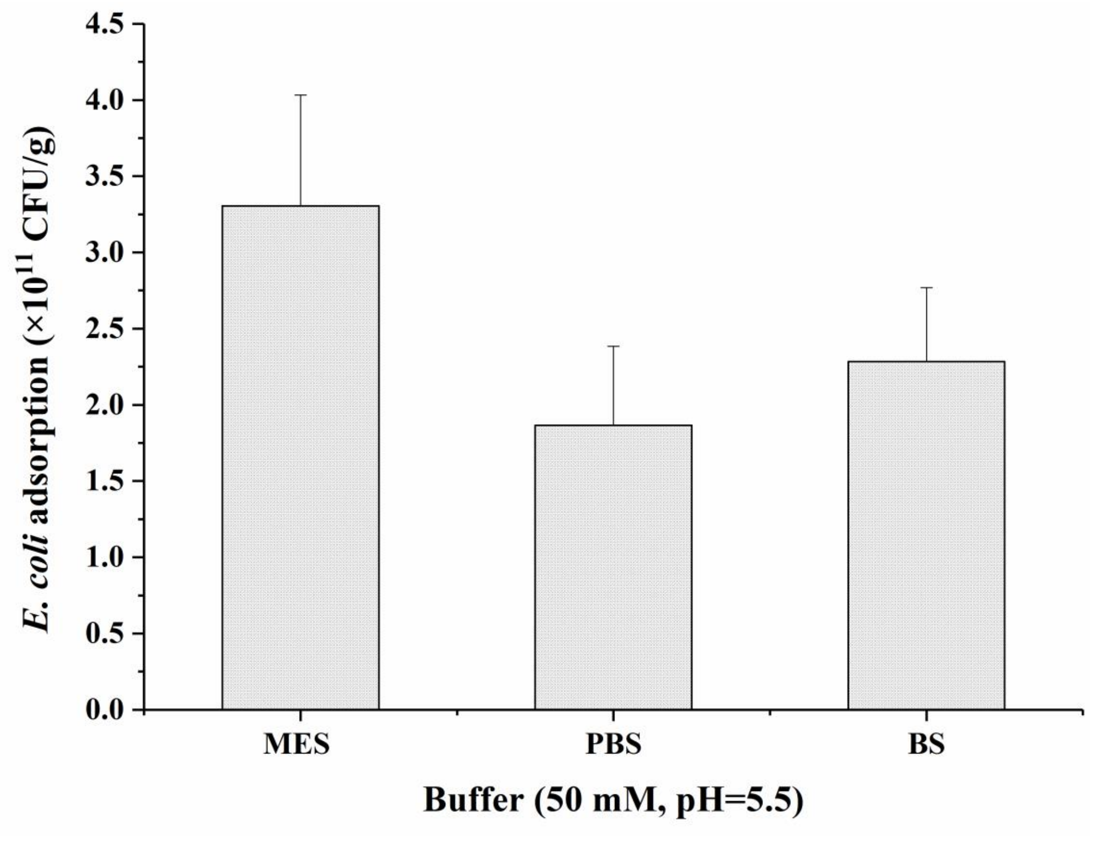

Figure S1: Effect of reaction buffers on adsorption of E. coli by SiO_2@NH_2@COOH@CST.

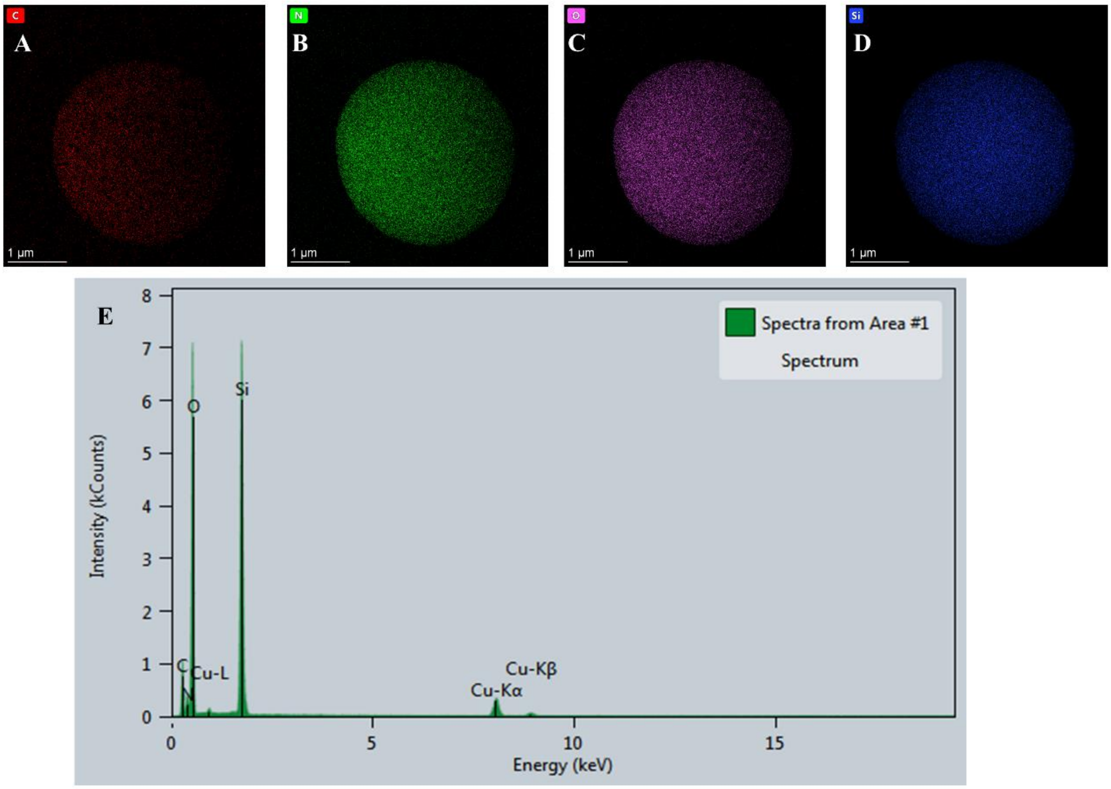

Figure S2: Element mapping of C (A), N (B), O (C) and Si (D) of SiO2@NH2@COOH@CST by TEM. EDS spectra of SiO2@NH2@COOH@CST depicting the expected elemental presence of C, N, Si, and O (E).

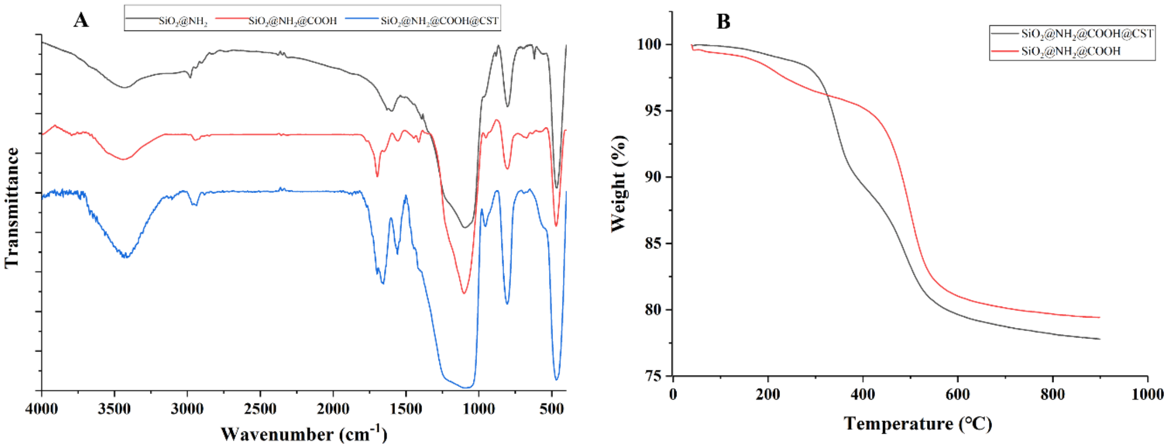

Figure S3: The FT-IR characterization of SiO2@NH2, SiO2@NH2@COOH and $SiO_2@NH_2@COOH@CST$ (A); the thermogravimetric analysis of $SiO_2@NH_2@COOH$ and $SiO_2@NH_2@COOH@CST$ (B).

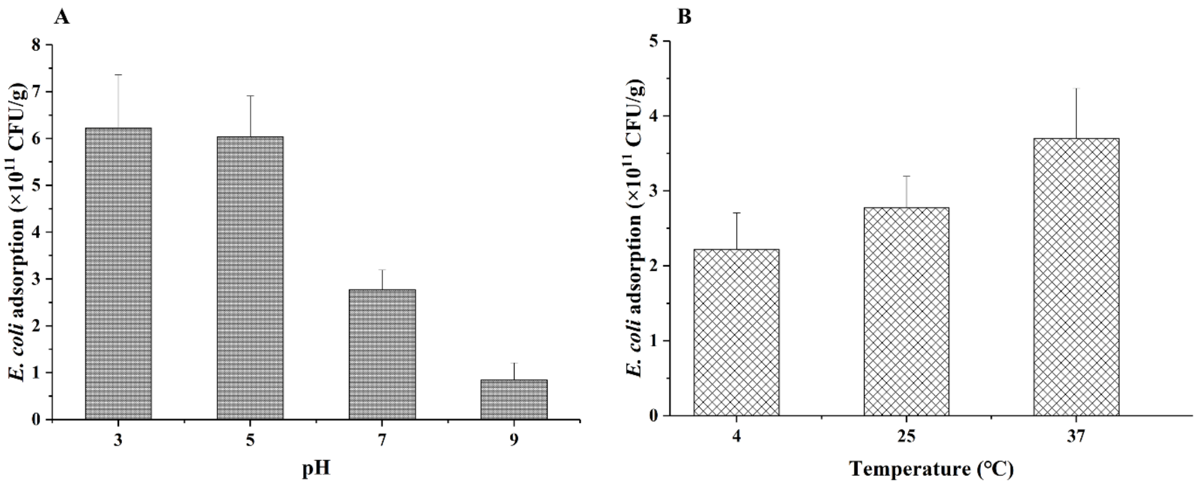

Figure S4: Effect of pH (A) and temperature (B) on the adsorption of E. coli by SiO_2@NH_2@COOH@CST.
